# Impaired energy expenditure following exposure to either DDT or DDE in mice may be mediated by DNA methylation changes in brown adipose

**DOI:** 10.1093/eep/dvae011

**Published:** 2024-08-23

**Authors:** Juliann A Jugan, Kyle B Jackson, Sarah E Elmore, Michele A La Merrill

**Affiliations:** Department of Environmental Toxicology, University of California, Davis, Davis, CA 95616, United States; Department of Environmental Toxicology, University of California, Davis, Davis, CA 95616, United States; Department of Environmental Toxicology, University of California, Davis, Davis, CA 95616, United States; Department of Environmental Toxicology, University of California, Davis, Davis, CA 95616, United States

**Keywords:** DNA methylation, brown adipose tissue, DDT, DDE, thermogenesis, oxidative phosphorylation, transcription, mTOR, steroidogenesis

## Abstract

The insecticide dichlorodiphenyltrichloroethane (DDT) and its persistent metabolite, dichlorodiphenyldichloroethylene (DDE), have been associated with increased adiposity and obesity in multiple generations of rodents and humans. These lipophilic pollutants accumulate in adipose tissue and appear to decrease energy expenditure through the impairment of thermogenesis in brown adipose tissue (BAT). We hypothesized that impaired thermogenesis is due to persistent epigenetic modifications of BAT. To address this, we exposed C57BL/6 J mice to DDT or DDE from gestational day (GD) 11.5 to postnatal day (PND) 5, evaluated longitudinal body temperature, and performed reduced representation bisulfite sequencing and RNA sequencing of BAT from infant and adult offspring. Exposure to DDT or DDE reduced core body temperature in adult mice, and differential methylation at the pathway and gene level was persistent from infancy to adulthood. Furthermore, thermogenesis and biological pathways essential for thermogenic function, such as oxidative phosphorylation and mechanistic target of rapamycin kinase (mTOR) signaling, were enriched with differential methylation and RNA transcription in adult mice exposed to DDT or DDE. PAZ6 human brown preadipocytes were differentiated in the presence of DDT or DDE to understand the brown adipocyte-autonomous effect of these pollutants. *In vitro* exposure led to limited changes in RNA expression; however, mitochondrial membrane potential was decreased *in vitro* with 0.1 µM and 1 µM doses of DDT or DDE. These results demonstrate that concentrations of DDT and DDE relevant to human exposure have a significant effect on thermogenesis, the transcriptome, and DNA methylome of mouse BAT and the mitochondrial function of human brown adipocytes.

## Introduction

Obesity has been rising worldwide [1]. For example, 42.5% of adults in the USA were estimated to be affected by obesity between 2017 and 2018 [2]. This increase in adiposity has not been limited to humans; 20 000 organisms across an additional 8 mammalian species, living in rural, urban, and controlled laboratory environments, all had increased body weight over the span of at least three decades [3]. Although increasing adiposity among humans and their associated mammals (e.g. cats, dogs, and rats) is generally attributed to changes in caloric intake and expenditure, this does not explain the increased fat and body mass observed among control group research mammals (e.g. monkeys, rats, and mice) [3]. An alternate explanation is that environmental chemicals are leading to increased adiposity across mammals.

Environmental toxicants, such as bisphenol A and phthalates, and some persistent organic pollutants (POPs) have been associated with increased risk of obesity and alterations in lipid metabolism [4]. As a proof of principle of the role of synthetic chemicals in contribution to weight gain and obesity, it has been well documented that numerous classes of pharmaceuticals, such as antipsychotics and glucocorticoids [5, 6], can lead to kilograms of weight gain within weeks of sustained use.

Despite restricted production and use of the insecticide dichlorodiphenyltrichloroethane (DDT) by the United Nations Stockholm Convention on POPs, exposure to DDT and its metabolite dichlorodiphenyldichloroethylene (DDE) is ongoing. For the purpose of clarity, we utilize “DDX” to indicate when the effects of DDT vs. DDE are not distinguished, as is common in whole organism studies due to the metabolism of DDT into DDE. Exposure to DDT and DDE is notably high among individuals residing in nations that are yet to ratify the Stockholm Convention, in nations continuing to manufacture or use DDT for control of malaria [7, 8], and in individuals migrating from such countries [9–11]. Due to their high lipophilicity, both POPs are stored for years in the adipose of humans and the human food supply [12, 13].

Meta-analyses of diverse prospective human cohorts that were conducted primarily after the ban of DDT indicate that exposure to both DDT and DDE contributes to the global rise in obesity [14, 15]. Furthermore, the association between DDT exposure and adiposity has also been observed across multiple generations in both humans and rats [16, 17]. For example, in the Child Health and Development Studies (CHDS) multigenerational human cohort, maternal serum concentrations of DDT were positively associated with increased body mass index and obesity across middle-aged daughters [18] and adult granddaughters [19]. While the CHDS did not collect data on sons and grandsons, meta-analyses do not reveal sex-specific effects of DDT or DDE despite their well-characterized effects on estrogen and androgen receptors [20]. The consistency of the positive association between DDT and DDE exposure and adiposity across generations and *in vitro* studies of multiple mammalian species substantiates the hypothesis that DDT and DDE are obesogens [14] although an alternative mechanism to sex hormone receptor effects remains to be elucidated.

Obesity arises when there is an imbalance in energy homeostasis, such as a shift toward reduced energy expenditure. A substantial proportion of endotherm daily calorie utilization includes the maintenance of body temperature through nonshivering thermogenesis [21]. Nonshivering thermogenesis is generated in brown adipocyte mitochondria by uncoupling respiration from the synthesis of adenosine triphosphate (ATP), an energy-rich molecule that can be used to fuel cellular processes [22]. Recently, it has been shown that brown adipose tissue (BAT) significantly contributes to energy expenditure in adult humans and mice [23–26]. Prior to these adult observations, it was thought that BAT activity was limited to the neonatal period.

Decreased body temperature and energy expenditure in adult mice exposed to perinatal DDX could be explained by decreased expression of genes that regulate thermogenesis in BAT, decreased sympathetic connectivity, and innervation of BAT [27, 28]. We hypothesize that direct exposure of brown adipocytes to DDX causes epigenetic and RNA expression changes with subsequent impaired thermogenesis in brown adipocytes independent of neurotoxicity.

In this study, we investigated the effect of perinatal DDT or DDE exposure on the transcriptome and DNA methylome of BAT from infant and adult female mice using RNA sequencing (RNA-seq) and reduced representation bisulfite sequencing (RRBS). The doses of DDT and DDE were selected to be directly relevant to human exposures and fall within the range of concentrations found in human sera collected in the 20th and 21st centuries [14, 15, 29–31]. An exposure between embryonic Day 11.5 and PND 5 was used to span the development of BAT [32, 33] and to include a window critical for metabolic programming, as observed in metabolic studies [34, 35] and through research on low doses of endocrine disruptors [36]. Additionally, this exposure period in mice is translatable to the development of tissue with high metabolic activity in humans, such as adipose, liver, and pancreas [37–39]. Female mice were chosen as the focus for this investigation because previous studies found increased adiposity associated with perinatal exposure to DDX in female humans [18] and mice [27] and decreased body temperature in female, but not male, mice perinatally exposed to DDX [28]. No clear sexual dimorphism has been found in outcomes related to obesity or diabetes in humans [40–43], and thus, our focus on female mice allows greater depth of research. Given the widespread changes to key biological pathways involved in energy expenditure seen in these transcriptomic and methylomic data, we then cultured human brown preadipocytes (PAZ6) in the presence of DDT or DDE over the period of adipogenic differentiation. The aim of this *in vitro* work was to determine the cell-autonomous effect of DDT or DDE on mitochondrial function and gene expression with respect to the pathways enriched in the mouse studies. Although we observed few changes in gene expression *in vitro*, we recorded a defect in mitochondrial membrane potential of both the DDT- and DDE-exposed PAZ6 cells.

## Results

### Perinatal DDX exposure reduced body temperature of mice into adulthood

**Figure 1.**
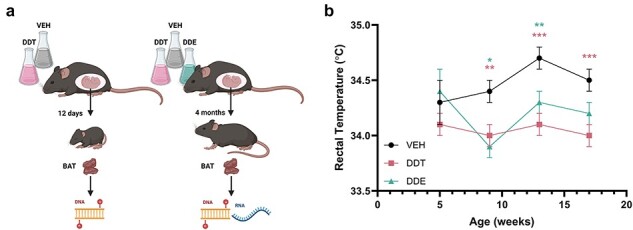
Mouse study design and DDX exposure phenotype. (a) Experimental design: nulliparous C57BL/6 J mice were mated and primigravid females were randomly assigned to an experimental group. Dams were orally gavaged from GD 11.5 to PND 5 with olive oil (VEH), a mixture of DDT or DDE. Offsprings from the infant mouse study were euthanized at PND 12, and BAT was collected for RRBS. Offsprings from the adult mouse study were euthanized at 4 months of age, and BAT was collected for RRBS and RNA-seq. (b) Rectal temperature measured at 5, 9, 13, and 17 weeks in female offspring from adult mouse study. Data shown are mean ± SEM, *n* = 14 (VEH), 15 (DDT), or 7 (DDE) female non-littermates. *P < .05, **P < .01, ***P < .001 DDX vs. age-matched VEH controls

Previous studies have shown that female mice perinatally exposed to a mixture of *o*,*p*'-DDT and *p*,*p*'-DDT, or DDE exhibited significant impairments in thermogenesis throughout maturation and into adulthood [27, 28]. Dams were exposed to a mixture of *o*,*p*'-DDT and *p*,*p*'-DDT (DDT), or DDE at a dose relevant to human exposure [29–31] from GD 11.5 to PND 5 (Fig. 1a). At 9 weeks of age, DDT and DDE exposure significantly reduced core body temperature, confirming the temperature impairment seen in previous studies. Core temperature impairment continued until Week 13 in DDE-exposed mice and through the end of the study (Week 17) in DDT-exposed mice (Fig. 1b), possibly indicating that DDT is more potent than DDE in impairing thermogenesis.

### Perinatal exposure to DDX led to differential gene methylation

**Figure 2.**
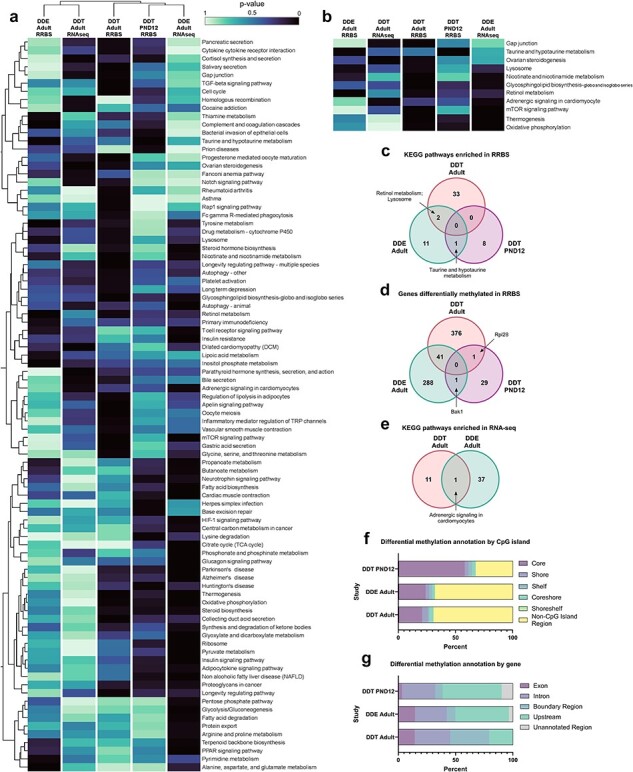
Enriched KEGG pathways in mouse studies. (a) Heatmap illustrating all KEGG pathways significantly enriched (*P *< .05) in at least one analysis and (b) selected KEGG pathways significantly enriched across more than one: age, exposure, or nucleic acid (RNA and DNA), based on RRBS or RNA sequencing (RNA-seq) of BAT from adult (DDT *n* = 16 DNA, *n* = 14 RNA; DDE *n* = 7 DNA, *n* = 5 RNA; VEH *n* = 14 DNA, *n* = 11 RNA) or infant female mice (tissue pool from 2–5 litter mates, DDT *n* = 12 DNA, VEH *n* = 12 RNA) perinatally exposed to either DDT or DDE, relative to VEH control. Gradient scale in (a) and (b) represents *P*-value. (c–e) Venn diagrams depicting significantly enriched KEGG pathways and differentially methylated genes in BAT from adult or infant female mice perinatally exposed to DDX relative to VEH control. Distribution of (f) differential methylation across the CpG island region and (g) the gene coding region of significant DMRs from the three RRBS mouse studies

To identify genes altered by perinatal DDX exposure, genome-wide differentially methylated regions (DMRs) in the intrascapular BAT of infant and adult mice were analyzed using RRBS and differential expression (DE) in BAT of adult mice was analyzed using RNA-seq. The amount of BAT in infant and adult mice did not vary between exposure groups at the time of collection (data not shown). When the Benjamini–Hochberg false discovery rate (FDR) correction was used to determine statistical significance (FDR < .05) of DMRs and DE, extensive DMRs, but no DE, were observed in BAT from mice exposed to DDT or DDE relative to control at the ages examined. Adult mice exposed to DDT had the greatest number of differentially methylated genes (FDR < .05, 418 genes) compared to adult mice exposed to DDE (FDR < .05, 330 genes) and infant mice exposed to DDT (FDR < .05, 31 genes, Fig. 2d, Supplementary Table S1). When we compared across treatments and ages, 41 genes were differentially methylated in BAT of adult mice exposed to either DDT or DDE (FDR < .05). Only one gene was commonly differentially methylated in infants exposed to DDT and in adults exposed to DDT, ribosomal protein L28 (*Rpl28*, FDR < .05, Fig. 2d, Supplementary Table S1). Similarly, only one gene was differentially methylated in infants exposed to DDT and adults exposed to DDE, bcL2 antagonist/killer 1 (*Bak1*, FDR < .05, Fig. 2d, Supplementary Table S1).

We next compared DMRs by gene region across treatments and mouse ages. BAT of DDT-exposed infant mice had a greater percentage of DMRs at CpG islands than DDT- and DDE-exposed adult mice, illustrating that DNA methylation signatures from toxicant exposure change over development (FDR < .05, Fig. 2f), yet the number of differentially methylated genes and pathways increased with age (FDR < .05, *P* < .05, Fig. 2a and b). Relative to adult mice with exposure either DDT or DDE, infant mice exposed to DDT had the greatest amount of DMRs upstream of the transcription start site (TSS). DDT-exposed adult mice had four times the amount of DMRs in boundary regions of genes (FDR < .05, Fig. 2g) where splicing can be regulated by methylation signatures [44].

### Perinatal DDX exposure led to persistent enrichment of the taurine and hypotaurine pathway with DNA methylation

To determine biological pathways persistently enriched by DDT or DDE exposure, DNA methylation in BAT of infant and adult mice was analyzed using Wilcoxon rank-sum tests (*P *< .05, Supplementary Table S2). Adult mice perinatally exposed to DDT had the greatest number of Kyoto Encyclopedia of Genes and Genomes (KEGG) pathways (*P* < .05, 35 pathways) enriched with DNA methylation compared to DDE-exposed adults (*P* < .05, 14 pathways) and infant mice exposed to DDT (*P* < .05, nine pathways, Fig. 2a and c). The taurine and hypotaurine metabolism pathway was enriched with DNA methylation across infant mice exposed to DDT and adult mice exposed to DDE (infant DDT *P *< .01, adult DDE *P *< .01, Fig. 2a, Supplementary Fig. S1, Supplementary Table S2). No other pathways were enriched across life stages.

### Perinatal DDX exposure enriched the adrenergic signaling pathway with RNA expression

Transcriptomic analyses using RNA-seq were run in BAT collected from adult mice exposed to DDT, DDE, or vehicle (VEH), and the greatest number of enriched KEGG pathways (*P* < .05, 38 pathways) was observed in DDE-exposed adult mice compared to DDT-exposed adult mice (*P* < .05, 12 pathways, Fig. 2a and e). Notably, both the adult DDT and adult DDE exposure groups showed enrichment of one pathway by RNA, the adrenergic signaling in cardiomyocyte pathway (DDT *P *< .05, DDE *P *< .05, Fig. 2e, Supplementary Fig. S2, Supplementary Table S2). Enrichment of RNA expression in the adrenergic signaling in cardiomyocytes pathway in adult mice exposed to DDT and DDE could suggest a DDE-specific effect on RNA expression because DDT-exposed mice were also exposed to DDE through the metabolism of DDT.

### Perinatal DDT exposure uniquely enriched the gap junction and ovarian steroidogenesis pathways with DNA methylation and RNA expression

**Figure 3.**
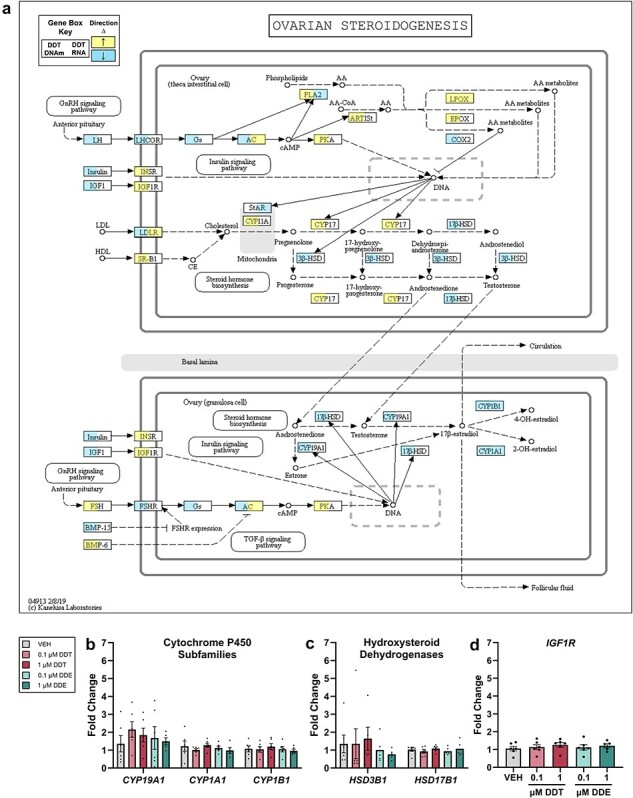
Enrichment of the ovarian steroidogenesis pathway in adult mice and related gene expression in human brown adipocytes. (a) KEGG pathway for ovarian steroidogenesis was enriched (*P* < .05) in BAT of adult female mice perinatally exposed to DDT where an increase in DNA methylation (left) or RNA expression (right) is represented by yellow and decrease is represented by blue. (b–d) Fold change in gene expression relative to *B2M* using RT-qPCR in differentiated PAZ6 human brown adipocytes exposed to dimethyl sulfoxide VEH or DDX (*n* = 6). Datapoints shown represent individual values ± SEM

To reveal the overlap of the KEGG pathways across all experimental conditions, we created a heatmap to compare enrichment that spanned nucleic acids (DNA and RNA), treatments, and mouse ages (*P* < .05, Fig. 2a). We found 11 pathways enriched across more than one of these conditions (*P* < .05, Fig. 2b, Supplementary Fig. S1–S5). Only the gap junction (adult, DDT: RNA *P *< .05, DNA *P *< .05, Supplementary Fig. S3) and ovarian steroidogenesis (adult, DDT: RNA *P *< .01, DNA *P *< .05, Fig. 2b, Fig. 3a, Supplementary Table S2) pathways were enriched across DDT exposure but not DDE exposure, suggesting that these pathways are uniquely impaired by DDT in BAT. None of the pathways were enriched by DDE for both DNA methylation and RNA transcription (Fig. 2b).

### Ovarian steroidogenesis pathway was enriched following perinatal DDX exposure

From the analysis of overlap in pathway enrichment across nucleic acids, treatments, and ages, we chose to focus on a subset of 11 pathways where enrichment occurred across DNA methylation and RNA expression (*P *< .05, Fig. 2b) for further investigation of *in vitro* model of brown adipocytes. This overlap across nucleic acids in murine BAT suggests a *cis*-regulatory role of DNA methylation with its expression to affect pathway function. To isolate the DDT vs. DDE effects, PAZ6 human brown preadipocytes were differentiated in the presence of either *p*,*p*'-DDT or *p*,*p*'-DDE at two concentrations, 1 µM and 0.1 µM, which were relevant to the circulating *p*,*p*'-DDT (0.144 µM) in the blood of DDT-treated dams and slightly greater than the circulating *p*,*p*'-DDE (0.007 µM) [27] and within human studies [14]. PAZ6 viability was not impacted by these concentrations of *p*,*p*'-DDT or *p*,*p*'-DDE (Supplementary Fig. S6). Once PAZ6 was fully differentiated and presented with characteristic browning and lipid accumulation (Supplementary Fig. S7), RNA and DNA were isolated for analysis. Because the sex of PAZ6 cells was relevant to our comparison to female mice, we determined that the PAZ6 preadipocyte cell line was donated by a male through the presence of sex-determining region Y gene (SRY) DNA (Supplementary Fig. S8).

BAT of DDT-exposed adult mice had enrichment of the ovarian steroidogenesis pathway across RRBS and RNA-seq analyses (DNA *P *< .05, RNA *P *< .01, Fig. 3a) but was not enriched in BAT of adult mice exposed to DDE or infant mice exposed to DDT (Supplementary Table S2). However, two pathways with related functions, the steroid biosynthesis and steroid hormone biosynthesis pathways, were enriched in BAT of DDE-exposed adult mice following RNA-seq analysis (*P *< .05, Supplementary Table S2). DDT and DDE have well-characterized agonism of estrogen and antagonism of androgen receptors [20], which could cause feedback in the steroidogenesis pathway. Within the brown adipocyte cell culture model, there was no significant change in the expression of cytochrome P450s, which catalyze metabolism of xenobiotic and endogenous substrates such as 17β-estradiol [45] and androstenedione [46]: cytochrome P450 family 19 subfamily A member 1 (*CYP19A1)*, cytochrome P450 family 1 subfamily B member 1 (*CYP1B1*), or cytochrome P450 family 1 subfamily A member 1 (*CYP1A1*) (Fig. 3b). There was also no significant change in RNA expression of hydroxy-delta-5-steroid dehydrogenase, 3 beta- and steroid delta-isomerase 1 (*HSD3B1*) and hydroxysteroid 17-beta dehydrogenase 1 (*HSD17B1*), regulators of androgen and estrogen synthesis, or insulin-like growth factor 1 receptor *IGF1R*, a receptor that functions to respond to stimuli of steroidogenesis and to maintain metabolic homeostasis in BAT [47] (Fig. 3c and d).

### mTOR signaling pathway was enriched following perinatal DDX exposure

**Figure 4.**
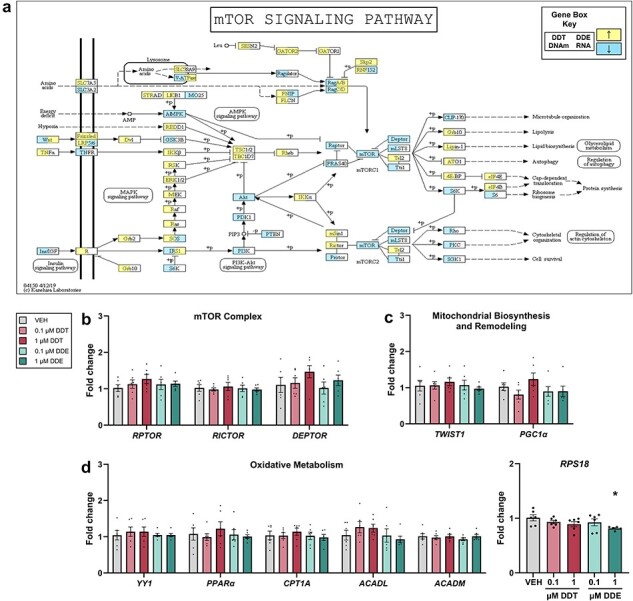
Enrichment of the mTOR signaling pathway in adult female mice and related gene expression in human brown adipocytes. (a) KEGG pathway for mTOR signaling was enriched (*P *< .05) in adult mice perinatally exposed to DDT (left) or DDE (right) where an increase in DNA methylation (left) or RNA expression (right) is represented by yellow and decrease is represented by blue. (b–e) Fold change in gene expression relative to *B2M* using RT-qPCR in differentiated PAZ6 human brown adipocytes exposed to VEH or DDX (*n* = 6). Datapoints shown represent individual values ± SEM

In BAT of adult mice, the mTOR pathway was significantly enriched with methylated DNA following DDT exposure (*P *< .01) and RNA following DDE exposure (*P *< .01, Fig. 4a, Supplementary Table S2). This pathway was not enriched in BAT with RNA following DDT exposure in adults, methylated DNA following DDE exposure in adults, or methylated DNA following DDT exposure in infants (Supplementary Table S2). Although mTOR signaling regulates adipose tissue development and thermogenesis [48–50], mTOR complex members, including the DEP domain containing MTOR interacting protein (*DEPTOR*), regulatory associated protein of MTOR, Complex 1 (*RPTOR*), and RPTOR independent companion of MTOR, Complex 2 (*RICTOR*) had no change in mRNA expression following DDT or DDE exposure throughout PAZ6 differentiation (Fig. 4b). There was also no change in expression of genes encoding proteins that interact with—or are downstream of—mTOR and also involved in oxidative metabolism [51], including peroxisome proliferative activated receptor, gamma, coactivator 1 alpha (*PPARGC1α*) [52, 53], twist family bHLH transcription factor 1 (*TWIST1*) [54], YY1 transcription factor (*YY1*) [55, 56], peroxisome proliferator activated receptor alpha (*PPARα*) [57], carnitine palmitoyltransferase 1A (*CPT1A*) [58], acyl-CoA dehydrogenase long chain (*ACADL*) [59], and acyl-CoA dehydrogenase medium chain (*ACADM*) [60] (Fig. 4c and d). We did, however, find that PAZ6 cells exposed to 1 μM DDE had a significant decrease (*P* < .05) in expression of ribosomal protein S18, (mRNA) *RPS18*, which facilitates protein synthesis downstream of the mTOR pathway (Fig. 4e).

### DDX exposure led to disruption of oxidative phosphorylation in vivo and in vitro

**Figure 5.**
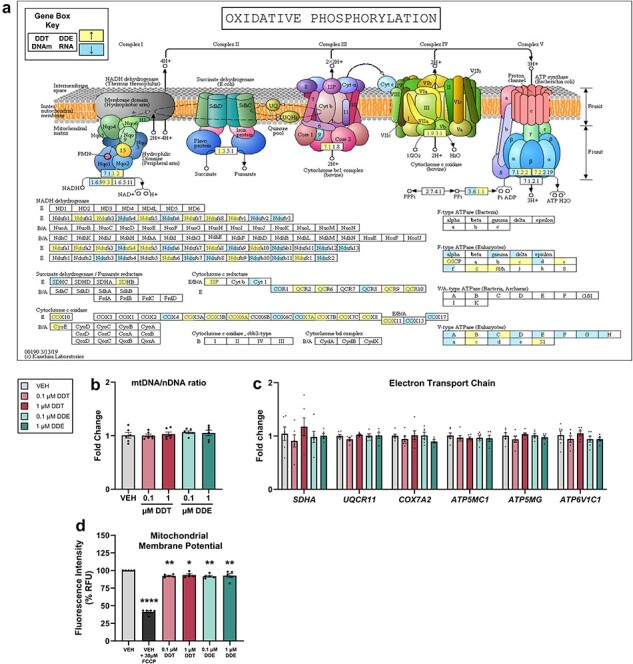
Enrichment of the oxidative phosphorylation pathway in adult female mice and validation in human brown adipocytes. (a) KEGG pathway for oxidative phosphorylation was enriched (*P *< .05) in adult mice perinatally exposed to DDT (left) or DDE (right) where an increase in DNA methylation (left) or RNA expression (right) is represented by yellow and decrease is represented by blue. (b) Relative ratio of mitochondrial DNA to nuclear DNA in PAZ6 cells exposed to dimethyl sulfoxide VEH or DDX (*n* = 6). (c) Fold change in gene expression relative to *B2M* using RT-qPCR in differentiated PAZ6 human brown adipocytes exposed to VEH or DDX (*n* = 6). (d) Mitochondrial membrane potential in PAZ6 cells exposed to VEH, FCCP, or DDX assessed by TMRE assay (*n* = 5). Data points represent the average percent relative fluorescent units per treatment for each plate. Statistical analyses were performed using one-way ANOVA. *P < .05, **P < .01, ****P < .0001 DDX vs. VEH with individual values or means ± SEM

The oxidative phosphorylation pathway was enriched in BAT of adult mice with both methylated DNA (DDT *P *< .01) and RNA (DDE *P* < .01, Fig. 5a) but was not enriched with DNA methylation from DDE-exposed adults or DDT-exposed infants or with RNA from DDT-exposed adults (Supplementary Table S2). Given these observations along with the essential role of oxidative phosphorylation in energy expenditure, we wanted to clarify the cell-autonomous impact of DDT or DDE on the mitochondria using PAZ6 cells differentiated in the presence of DDT or DDE. We found that the ratio of mitochondrial DNA to nuclear DNA did not vary across treatments (Fig. 5b), indicating that changes in mitochondrial abundance were not the underlying cause of oxidative phosphorylation pathway enrichment. We then analyzed the transcript level of genes encoding key electron transport chain proteins that were enriched in the oxidative phosphorylation pathway in mouse BAT (*P* < .05, Fig. 5a); however, no changes in expression of these genes were evident *in vitro* (Fig. 5c).

To determine whether DDT or DDE treatment impaired mitochondrial function within brown adipocytes in a cell-autonomous manner, we measured mitochondrial membrane potential of PAZ6 cells following treatments. A decrease in mitochondrial membrane potential (i.e. depolarization) was observed in mature PAZ6 cells at all concentrations of DDT and DDE (*P *< .05, Fig. 5d), consistent with literature demonstrating the reduction in ATP production capacity through oxidative phosphorylation by DDT and DDE *in vitro* [61, 62].

### Thermogenesis pathway was enriched following perinatal DDX exposure

**Figure 6.**
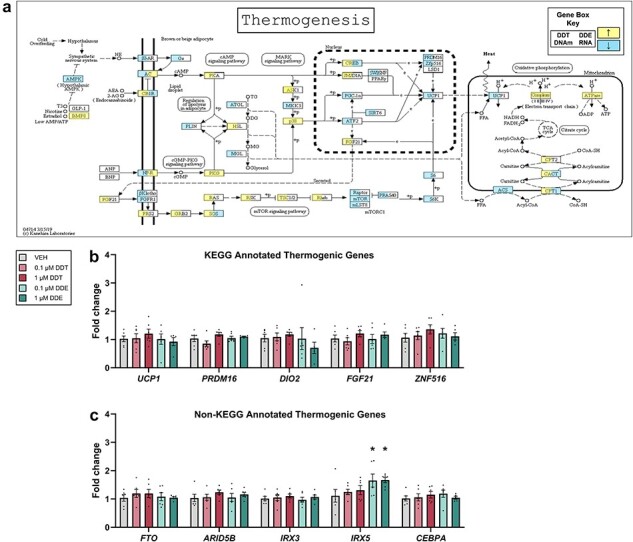
Enrichment of the thermogenesis pathway in adult mice and related gene expression in human brown adipocytes. (a) KEGG pathway for thermogenesis was enriched (*P *< .05) in adult female mice perinatally exposed to DDT (left) or DDE (right) where an increase in DNA methylation (left) or RNA expression (right) is represented by yellow and decrease is represented by blue. (b–c) Fold change in gene expression relative to *B2M* using RT-qPCR in differentiated PAZ6 human brown adipocytes exposed to VEH or DDX (*n* = 6). Statistical analyses were performed using one-way ANOVA. *P < .05 DDX vs. VEH with individual values ± SEM

In BAT of adult mice, the thermogenesis pathway was enriched for DNA methylation with DDT exposure (*P *< .01) and RNA transcription with DDE exposure (*P *< .01, Fig. 6a, Supplementary Table S2), in alignment with decreased body temperature in mice with either exposure (Fig. 1b). However, the thermogenesis pathway was not enriched with DNA methylation in adult mice exposed to DDE or infant mice exposed to DDT, nor was it enriched with RNA in DDT-exposed adult mice (Supplementary Table S2). Genes key to brown adipocyte function and enriched following perinatal DDX exposure *in vivo*, including uncoupling protein 1 (*UCP1*) [22], PR/SET domain 16 (*PRDM16*) [63], iodothyronine deiodinase 2 (*DIO2*) [64], fibroblast growth factor 21 (*FGF21*) [65], and zinc finger protein 516 (*ZNF516*) [66] had no significant changes in gene expression *in vitro* after PAZ6 exposure to DDT or DDE (Fig. 6b). In addition to the KEGG-annotated genes, we were interested in the expression of genes relevant to human adipose thermogenesis not yet annotated in the KEGG thermogenesis pathway, e.g. fat mass and obesity-associated gene *FTO* (*FTO*), associated AT-rich interaction domain 5B (*ARID5B*), iroquois homeobox 5 (*IRX5*), and iroquois homeobox 3 (*IRX3*) [67]. BAT of adult mice showed differential methylation of IRX3 following either DDT or DDE exposure and differential methylation of IRX5 following DDE exposure (FDR < .05, Supplementary Table S1). *IRX5* expression was significantly increased in PAZ6 cells exposed to DDE (*P* < .05, Fig. 6c), consistent with impaired thermogenesis [67].

## Discussion

We investigated the effect of perinatal DDX exposure on the transcriptome and DNA methylome of adult and infant mouse BAT. Consistent with previously published studies showing impaired body temperature following DDX exposure [27, 28, 68], body temperature was decreased in mice from peripubertal development into adulthood. Changes to the transcriptome and DNA methylome were widespread and encompassed key biological pathways involved in energy expenditure, supporting our primary hypothesis that perinatal exposure to either DDT or DDE would have an epigenetic effect on thermogenesis. Human brown adipocytes cultured in DDT or DDE over the course of differentiation did not show changes in the expression of all genes enriched in mouse studies; however, the exposed human brown adipocytes exhibited decreased mitochondrial membrane potential and decreased expression of *IRX5*, suggesting that there are cross-species effects of DDT and DDE autonomous to brown adipocytes. DNA methylation persisted across ages in pathways essential to energy homeostasis, as evident by enrichment of the taurine and hypotaurine metabolism pathway with DNA methylation in BAT from infant and adult mice exposed to DDX. Impaired taurine metabolism may explain DDX effects on brown adipose thermogenesis, obesity, and insulin resistance given that taurine supplementation in mice decreased diet-induced obesity while increasing thermogenesis and the expression of thermogenic genes in BAT [69–71]. While such *in vivo* observations could be related to taurine’s suppression of sympathetic activity to preserve insulin sensitivity reported as in numerous rodent models, brown adipocyte-autonomous effects of taurine, DDT, and DDE have also been observed [72]. For example, similar to the mitochondrial membrane potential depolarization) observed in mature PAZ6 cells exposed to all concentrations of DDT and DDE, taurine loss in brown adipocytes decreased the mitochondrial oxygen consumption rate and UCP1 protein expression [73]. The persistent effect of early-life DDE exposure on the DNA methylome was also demonstrated in rats. A study performed by Wang et al. found that susceptibility to diabetes into adulthood and in subsequent generations increased with prenatal DDE exposure [74]. This incidence of diabetes was mediated by changes in DNA methylation of sperm and liver; however, KEGG enrichment analyses performed on sperm did not show enrichment of the taurine and hypotaurine metabolism pathway [74]. Transgenerational inheritance of epigenetic modifications also led to increased obesity of F3 rats, where the F1 generation was exposed to DDX *in utero*. Over 150 genes associated with the DMRs of sperm DNA were categorized under the broad KEGG grouping of metabolic pathways, yet further pathway details were not shown [16]. Given that these rat studies did conduct pathway analysis of DNAm in sperm or examine BAT, future studies will need to confirm our reported effects of DDX on taurine metabolism in other metabolically active tissues.

DDT and DDE are known ligands of the estrogen [75, 76] and androgen receptors [77], and a limited, but supportive, number of studies have described the disruption of steroidogenesis by DDT and DDE [78–80]. Steroidogenesis, such as estrogen synthesis, has been well documented in white adipose tissue [81], though seldom studied in BAT [82, 83]. It remains unclear whether sex specific aspects of thermogenesis include adipocyte-autonomous effects in addition to endocrine effects [84, 85]. There was no significant change in expression of genes encoding steroid biosynthesis (e.g. *CYP19A1, HSD3B1*, and *HSD17B1*) in human brown adipocytes exposed to DDT or DDE; however, enrichment of these and other genes in steroid biosynthesis pathways following *in vivo* exposure to DDX may contribute to the female sex–specific effects of DDX on impaired thermogenesis in mice [27, 28].

The extensive alterations in DNA methylation and RNA levels of the mTOR pathway by DDX *in vivo* suggested that DDX could modify mTOR enhancement of protein synthesis, lipid metabolism, thermogenesis, and/or attenuation of insulin signaling [49, 86, 87]. A possible brown adipocyte-autonomous effect of DDE on the mTOR pathway was revealed by the reduced expression of a key ribosomal subunit in PAZ-6 cells. We were unable to find studies investigating the effect of DDT or DDE on mTOR function in BAT. However, in myoblasts, which share the same stem cell lineage as brown preadipocytes [88], the protein ratio of mTOR to phosphorylated mTOR was increased and myotube formation, indicative of successful differentiation by mTOR, decreased following either DDT or DDE exposure [89]. Our results reveal that DDX exposure *in vivo* can directly alter the DNA methylation of key mTOR signaling genes, providing a possible mechanism to the aberrant lipid, glucose, and energy metabolism seen in association with DDX exposure.

The oxidative phosphorylation pathway enriched by DDX *in vivo* along with depolarization of the mitochondrial membrane following DDT and DDE exposure of human brown adipocytes *in vitro* is consistent with rat liver mitochondria exposed to DDT, and DDE exhibited mitochondrial membrane depolarization and impaired repolarization when stimulated with succinate [61, 62]. Indeed, depolarization of the inner mitochondrial membrane is supported by extensive evidence of DDT and DDE inhibiting numerous electron transport chain complexes across tissues (e.g. hepatic, cardiac, and brain) and species (rats, cattle, and human) [61, 62, 87, 90, 91]. While depolarization of the inner mitochondrial membrane can reflect thermogenesis and/or ATP depletion [90], numerous lines of evidence support the later and not the former. For example, DDT targeted ATP synthase (Complex V) in many rat tissues and inhibited ATP synthase and turnover in human hepatocytes [90, 91]. We are unaware of DDT increasing thermogenesis other than tremorgenic levels [92]. Although our study of mitochondrial membrane potential was conducted on whole cells, mtDNA quantification indicated no difference in mitochondrial abundance and hence no effect of DDX exposure on browning of adipocytes.

Body temperature was reduced, and the thermogenesis pathway was enriched by DDX *in vivo*. However, these *in vivo* observations were not consistent with *in vitro* expression of genes annotated in the KEGG pathway for thermogenesis. This apparent discrepancy could arise from DDX toxicity to the sympathetic nervous system upstream of brown adipose [28] or sex-specific differences across the models we used (female mice, male PAZ6 cells) given that in a study of women’s sera, DDE was associated with the carnitine shuttle, an important portion of the thermogenesis pathway [87]. However, not all thermogenesis genes are annotated to the KEGG thermogenesis pathway. For example, the FTO SNP consistently associated with obesity in human studies leads to increased expression of *IRX5* and decreased thermogenesis [67, 93]. Consistent with some component of impaired thermogenesis resulting from direct toxicity of DDE to the brown adipocyte, expression of *IRX5* increased with DDE exposure in PAZ6 cells. This does not rule out the potential for larger effects of DDX to thermogenic genes in the context of a sympathetic nervous system and/or brown adipocytes from human females. Further studies are required to examine the potential relationship between DDX-induced DNA methylation and brown adipocyte thermogenesis.

It is surprising that we observed functional changes across *in vivo* and *in vitro* models with exposure to DDX, yet limited alterations to RNA expression by DDX *in vivo* and *in vitro*. We did not find significant DE *in vivo*, but only found significant changes in RNA expression at the KEGG pathway level *in vivo* and in a limited number of genes by qPCR *in vitro* despite the (i) decreased body temperature in the adult mice perinatally exposed to DDX here and elsewhere at numerous ages [27, 28], (ii) significantly reduced expression of multiple pro-thermogenesis genes in BAT in 9-month-old mice exposed to DDT using the same exposure protocol with qPCR [27], and (iii) depolarized mitochondrial membrane potential following DDT or DDE exposure *in vitro*. These results could mean that (i) RNA expression is not a reliable readout of the DDX-induced impaired thermogenesis in BAT at the times measured, (ii) RNA-seq was not sensitive enough to detect quantitative DE, (iii) transcript-level changes by DDX require a whole organism and cultured brown adipocytes do not exhibit the same phenotype as BAT from developmentally exposed mice, and/or the DDX-induced impairment of thermogenesis is (iv) sex- and/or (v) species- specific. Conversely, changes to DNA methylation in BAT samples were observable at both the pathway and gene level, following DDT or DDE developmental exposures and across ages of mice. PAZ6 brown adipocytes express similar brown markers and adaptive mechanisms to murine BAT [94–96], yet whether effects of DDT and DDE on DNA methylation in mouse BAT extend to cultured human adipocytes was out of the scope of this analysis. Women have greater BAT activity than men [97–99]; however, the activity of cultured brown-like adipocytes did not differ between sex despite sex differences in UCP1 RNA expression [100]. This suggests that sex differences in BAT activity are not brown adipocyte-autonomous, and thus, *in vitro* analysis of brown adipocytes is not suitable for analysis of potential sex differences. Additionally, we cannot rule out a direct genetic effect of DDX on brown adipose because there is moderate evidence that DDT exposure has mutagenic effects on mammalian cells and tissues [20] and deep sequencing for *de novo* mutations was outside of the scope of this work. Future studies of transcriptional changes, genotoxicity, and DNA methylation in BAT collected from humans with developmental exposure to DDT and/or DDE could address many of these unanswered questions.

DDT and DDE join other developmental conditions such as maternal diet [101, 102] and increased glucocorticoid levels [103] that have epigenetically altered BAT of offspring. To our knowledge, this is the first study to examine the relationship between exposure to DDX and DNA methylation changes during development of brown adipose *in vivo*. Additionally, we are not aware of other publications using a human brown adipocyte cell line to investigate the effect of DDT or DDE exposure on energy expenditure. Our data show that perinatal DDX exposure has a significant effect on the body temperature, transcriptome, and DNA methylome of mouse BAT and that DDT and DDE impair mitochondrial membrane potential in human brown adipocytes; all these effects were at doses relevant to the current human condition. The impairment of thermogenesis and enrichment of key metabolic pathways across DDX-induced differential gene expression and methylation, life stage, and exposure group further support the hypothesis that exposure to either DDT or DDE is deleterious to energy expenditure. Further research is required to pinpoint the mechanism behind the impairment in thermogenesis; however, these data are consistent with DDX exposures contributing to obesity by targeting various mitochondrial functions.

## Methods

### Chemicals

The DDT mixture used in dosing mice was a combination of 22.8% *o*,*p*'-DDT (100% purity neat, AccuStandard, New Haven, CT) and 77.2% *p*,*p*'-DDT (98.5% purity neat, AccuStandard), hereafter referred to as DDT, to mimic the commercial formulation utilized before the DDT ban in the USA. The DDE dose comprised only *p*,*p*'-DDE (100% purity neat, AccuStandard). Chemicals were dissolved at concentrations of 1.7 mg/kg (DDT) and 1.31 mg/kg (DDE) in a VEH of organic extra virgin olive oil from Italian grown olives (Nugget Markets, Woodland, CA)*. p*,*p*'-DDT (98.2% purity neat, AccuStandard) and *p*,*p*'-DDE (100% purity neat, AccuStandard) for use with cell culture were dissolved in 100% dimethyl sulfoxide (DMSO) (Sigma-Aldrich, St. Louis, MO).

### Animal handling and exposures

Nulliparous male and female 8-week-old C57BL/6 J mice were acclimated for at least 1 week following delivery from the vendor prior to breeding (Jackson Laboratories, Sacramento, CA). Pregnancy was determined by the presence of a postcoital vaginal plug. An experimental group was assigned randomly to each primigravid female based on random number generation and *a priori* decisions to maximize the number of litters per VEH and DDT treatment. Our primary objective was to study the effect of DDT exposure; thus, only mice in the cohort of adult offspring were perinatally exposed to DDE. Mice were exposed to DDT (0.39 mg of *o*,*p*'-DDT/kg body weight and 1.31 mg of *p*,*p*'-DDT/kg body weight) or DDE (1.31 mg of *p*,*p*'-DDE/kg body weight) at a human-relevant dose [14, 27] or certified organic olive oil (VEH) by oral gavage (10 ml/kg body weight) from GD 11.5 to PND 5. This dosing period was selected to span adipose tissue ontogenesis [104, 105]. Litters were culled to six random pups while maximizing the number of females at PND 5 to normalize the lactational transfer of DDT and DDE and maternal behavior effects, and avoid overnutrition [106]. In the infant mouse study, VEH- and DDT-exposed mice had mean (standard error, SE) litter sizes of 6.89 (0.38) and 7.45 (0.35), respectively (*P* = .28). In the adult study, VEH-, DDE-, and DDT-exposed mice had mean (SE) litter sizes of 8.40 (0.31), 8.25 (0.49), and 8.08 (0.28), respectively (*P* = .80 DDE vs. VEH and *P* = .46 DDT vs. VEH).

For the infant mouse study, female pups (*n* = 20 DDT litters, *n* = 22 VEH litters) were fasted 1–2 h and euthanized at PND 12 by exsanguination under isoflurane anesthesia. Intrascapular BAT was collected and pooled by litter (2–5 female pups) before being flash frozen in liquid nitrogen and stored at −80°C.

For the adult mouse study, pups were weaned at PND 21. Core body temperature of female mice was measured rectally at a depth of 5 mm using a RET-4 thermocouple probe (Physitemp, Clifton, NJ). Measurements were taken at 5 weeks of age and repeated every 4 weeks. One 4-month-old female from each litter (*n* = 16 DDT litters, *n* = 7 DDE litters, n = 14 VEH litters) was euthanized by exsanguination under isoflurane anesthesia. Intrascapular BAT was collected, flash frozen in liquid nitrogen, and stored at −80°C.

Food (Purina diet 5053) and water were provided *ad libitum*. Weaned mice were housed in ventilated single-sex cages with 2–5 mice, 12-h photoperiods, and 40%–50% humidity. Mice were maintained in a facility fully accredited by the American Association for the Accreditation of Laboratory Animal Care. Experiments were conducted according to the National Institutes of Health guide for the care, and use of laboratory animals and procedures were approved by the UC Davis Institutional Animal Care and Use Committee.

### DNA and RNA isolation

Frozen mouse BAT was pulverized with a mortar and pestle. DNA from adult and infant mice and RNA from adult mice were extracted using an AllPrep DNA/RNA/Protein Mini Kit (Qiagen, Germantown, MD). DNA was removed from RNA samples using DNase (New England Biolabs), and yield of DNA and RNA was determined using a Qubit 3.0 Fluorometer (Thermo Fisher Scientific Inc.). Nucleotide purity and quality were assessed using a NanoQuant Plate (Tecan, Männedorf, Switzerland) and micro-capillary gel electrophoresis (Fragment Analyzer, Agilent). Isolated DNA and RNA were stored at −80°C.

### RRBS library preparation, sequencing, and analysis

To prepare the RRBS libraries, DNA isolated from BAT was digested with MspI enzyme (New England Biolabs, Ipswich, MA), blunt-end fragments were repaired (NEB Next End Repair Module, New England Biolabs), and 3ʹ ends were adenylated with Klenow Exo Fragments (New England Biolabs). Methylated adaptor oligos (Integrated DNA Technologies, San Diego, CA) were ligated onto the DNA, and magnetic AmPure XP beads (Beckman Coulter Inc, Brea, CA) were used to select fragment sizes between 150 and 180 base pairs. Samples underwent bisulfite conversion (EZ DNA Methylation Gold Kit, Zymo Research Inc, Irvine, CA) and were amplified by polymerase chain reaction (PCR) for 15 cycles. The samples were then barcoded, multiplexed, and run with an average of 30 million reads mapped per sample. Samples were run on an Illumina HiSeq4000 with single-end 50 bp sequencing (infant mice) or single-end 100-bp sequencing (adult mice) by the University of California, Davis DNA Technologies and Expression Analysis Core.

Analysis of adult and infant RRBS libraries was performed in R (www.r-project.org) by the University of California, Davis Bioinformatics core. Sequenced single-end reads of 100 bp (adult mice; *n* = 16 DDT, *n* = 7 DDE, *n* = 14 VEH) and 50 bp (infant mice; *n* = 12 DDT, *n* = 12 VEH) were trimmed using Scythe [107]. Sickle [108] was used to trim bases with a Phred quality threshold below 30, and reads less than 30 bp were discarded. Reads that successfully passed quality control using FastQC were aligned to *Mus musculus* reference genome GRCm38 with Bismark version 0.15.0 [109] and Bowtie 2 [110] as the underlining aligner using -D 20 and -R 3 parameters to increase the chance for finding an alignment.

### RNA-seq library preparation, sequencing, and analysis

The RNA-seq library was prepared using RNA from BAT of the adult mice (NEBNext Ultra Directional RNA Library Prep Kit for Illumina, NEBNext Poly(A) mRNA Magnetic Isolation Module, New England Biolabs). Briefly, oligo beads removed rRNA through Poly(A) enrichment, followed by purification and fragmentation. The first strand of cDNA was synthesized with random hexamers, and the second strand was then generated. Following completion of terminal repair, poly-adenylation, and sequencing adaptor ligation, size selection and PCR enrichment (15 cycles) were completed with the resulting cDNA library. Quality of the 150-bp cDNA strands was assessed using an Agilent 2100 Bioanalyzer (HS RNA Assay, Agilent, Santa Clara, CA) before barcodes were added. Samples were sequenced on an Illumina HiSeq4000 with single-end 100-bp sequencing by the University of California, Davis DNA Technologies and Expression Analysis Core. The RNA-seq library of adult mice (*n* = 14 DDT, *n* = 5 DDE, *n* = 11 VEH) was filtered prior to analysis by the University of California, Davis Bioinformatics Core, to exclude genes with expression <2 counts per million, which resulted in 17 302 genes analyzed for DE. In R, sequenced reads were trimmed using Scythe [107] and Sickle [108], quality control was evaluated with FastQC, and the remaining reads were aligned to the GRCm38 reference genome using the Tophat and Cufflinks pipeline [111].

### Pathway analysis

Enrichment analyses for KEGG pathways [112] were performed for differential gene expression and DNA methylation (DNAm). The KEGGREST Bioconductor package in R [113] was used to identify pathway enrichment by Wilcoxon rank-sum tests, which tested that the *P*-values from the gene-level analyses between DDT or DDE treatment and VEH treatment were smaller than those genes not in the pathway.

### Brown adipocyte cell culture

PAZ6-immortalized human brown preadipocytes [94] were purchased from Applied Biological Materials (ABM, BC, Canada). PAZ6 preadipocytes were maintained according to ABM company protocol, in a humidified atmosphere at 5% CO_2_ and 37°C, with a culture medium of Pregrow IV (ABM) supplemented with 8% fetal bovine serum (FBS) (Gemini BioProducts, Sacramento, CA), 15 mM 4-(2-hydroxyethyl)-1-piperazineethanesulfonic acid (HEPES) (Gibco, Thermo Fisher Scientific Inc., Waltham, MA), 1% GlutaMAX (Gibco), and 1% Penicillin/Streptomycin (Gibco). For induction of differentiation into brown adipocytes, cells were seeded in plates coated with Applied Extracellular Matrix (ABM) at a density of 10 000 cells/cm^2^ and grown in culture medium to confluence. Confluent cells were then incubated for 4 days in a differentiation medium of Dulbecco’s Modified Eagle Medium F12 (DMEM/F12) (Gibco) with the addition of 5% FBS, 1% penicillin/streptomycin, 500 nM human insulin (Sigma-Aldrich), 1 nM triiodothyronine (Sigma-Aldrich), 1 µM pioglitazone (Sigma-Aldrich), 33 µM biotin (Sigma-Aldrich), 100 nM dexamethasone (Sigma-Aldrich), 0.25 mM 3-isobutyl-1-methylxanthine (IBMX) (Sigma-Aldrich), and 17 µM pantothenate (Research Products International, Mt Prospect, IL). For the final 10 days of differentiation, cells were provided differentiation medium without IBMX. During the 14-day differentiation period, medium was changed every 48 h. All cells except those used for Oil Red O staining were treated with concentrations of *p*,*p*'-DDT (0.1 µM, 1 µM) or *p*,*p*’-DDE (0.1 µM, 1 µM) relevant to human exposure [14] or 0.01% DMSO (VEH) beginning with the first supplemented media and continuing throughout differentiation.

### Oil Red O lipid stain

Intracellular lipid content of PAZ6 cells was visualized using an Oil Red O stain. Cells were seeded into 12-well plates (Corning) and cultured in differentiation media. At selected time points, cells were fixed in 10% neutral buffered formalin (Thermo Fisher Scientific Inc.) at room temperature for 1 h. Plates were washed with deionized (DI) H_2_O and fully dried. Fixed cells were then incubated for 30 min in a 0.3% Oil Red O (Sigma-Aldrich) solution and rinsed with DI H_2_O. Stained cells were imaged at 200× using an Olympus CKX53 culture microscope (Center Valley, PA).

### Trypan blue exclusion assay

Cell viability was assessed using a trypan blue exclusion assay. PAZ6 cells were seeded into a 96-well plate (Corning) and exposed to DDT, DDE, or VEH over differentiation. On Day 14 of differentiation, cells were dissociated from the plate using a 0.05% trypsin-ethylenediaminetetraacetic acid solution (Gibco) and the reaction was quenched in DMEM/F12 with 5% FBS. An aliquot of the cell suspension was removed, and a 0.4% Trypan Blue solution (Thermo Fisher Scientific Inc.) was added at a 1:1 dilution. Cells were incubated in the dye for 3 min and promptly counted using a Cellometer Auto T4 Cell Counter (Nexcelom Bioscience, Lawrence, MA). Cells stained by trypan blue were marked nonviable. Data are reported as percent cell death, where percent cell death = (total number of viable cells per ml/total number of cells per mL) × 100%.

### Brown adipocyte DNA/RNA isolation

Frozen, fully differentiated PAZ6 cells were lysed in six-well plates (Corning, Concord, NC), and DNA and RNA were extracted using an AllPrep DNA/RNA Mini Kit (Qiagen). gDNA contamination of RNA samples was avoided with RNase-Free DNase (Qiagen). DNA and RNA yield was determined using a Qubit 3.0 Fluorometer (Thermo Fisher Scientific Inc.).

### Gene expression

PAZ6 RNA (1 μg) was synthesized into cDNA using an Invitrogen SuperScript VILO cDNA Synthesis Kit (Thermo Fisher Scientific Inc). Quantitative real-time PCR (RT-PCR) was performed using Power SYBR™ Green PCR Master Mix (Thermo Fisher Scientific Inc.) on a QuantStudio 3 Real-Time PCR System (Thermo Fischer Scientific Inc.). Oligonucleotide primers were manufactured by Integrated DNA Technologies (Coralville, IA; Supplementary Table S3). The 2^-ddCT^ method was used to calculate fold change in gene expression relative to beta-2-microglobulin (*B2M*) as the endogenous control [114].

### Relative mtDNA ratio and sex determination

PAZ6 DNA was diluted to 10 ng/µl in nuclease-free water. Each reaction contained 20 ng of DNA and was carried out using Power SYBR™ Green PCR Master Mix (Thermo Fisher Scientific Inc.) on a QuantStudio 3 Real-Time PCR System (Thermo Fischer Scientific Inc.). Expression of SRY is reported as raw *C*_t_ value and compared to DNA expression from control samples (10 ng/µL DNA) of known human male (HEPG2) [115] and female (MDA-MB-231) [116] cells. The 2^-ddCT^ method was used to calculate fold change in mitochondrial 16S ribosomal RNA (MT-RNR2) expression relative to B2M as the endogenous control [114].

### Measurement of mitochondrial membrane potential

Mitochondrial membrane potential was measured in PAZ6 cells using the fluorescent dye tetramethylrhodamine ethyl ester (TMRE) (Thermo Fisher Scientific Inc.). PAZ6 cells were seeded in black-walled 96-well plates (Greiner Bio-One Inc., Monroe, NC) and fully differentiated in the presence of DDT, DDE, or VEH. On the final day of differentiation, media were removed and cells were incubated at 37°C and in 5% CO_2_ for 30 min in DMEM/F12 with their respective DDX treatment, VEH, or a depolarization control of 30 µM carbonyl cyanide 4-(trifluoromethoxy) phenylhydrazone (FCCP) (Sigma-Aldrich). Cells were then incubated at 37°C and in 5% CO_2_ for an additional 30 min in DMEM/F12 with their respective DDX treatment or VEH and a final concentration of 100 nM TMRE. After incubation, cells were washed in warmed phosphate buffered saline (PBS) (Gibco) and fluorescence was read at an *E*_x_/*E*_m_ value of 530/580 nm in an Infinite M200 Pro plate reader (Tecan, Männedorf, Switzerland). Relative fluorescence was calculated using the following equation: relative fluorescence signal (%) = (sample group fluorescence/VEH group fluorescence) × 100%.

### Statistical analyses

All datapoints are representative of the mean ± standard error. The least square means differences between categorical effects of treatment data were used to analyze the body temperatures of mice at each age and litter sizes because data were normally distributed (PROC GLM, SAS v9.4, Statistical Analysis System Institute, Cary, NC). A differential methylation analysis package was used in the analysis of DMRs of mouse BAT where gene regions were defined by their relative distances from the TSS and ranged from 29 to 219 bp as a result of our MspI fragment-based methylome [117]. Significance (FDR < .05) between exposure and VEH was determined by an Analysis of Variance (ANOVA) *F* test and Benjamini–Hochberg FDR correction. The analysis package limma-voom [118] and the Benjamini–Hochberg Procedure were used to determine significance (FDR < .05) in DE of genes in mouse BAT between exposure and VEH groups. *In vitro* data from gene expression and TMRE experiments were compared using a one-way ANOVA followed by Dunnett’s multiple comparisons test in GraphPad Prism (Version 10.0.0, GraphPad Software, Boston, MA) to determine significance (*P* < .05). Venn diagrams were created using Venny [119] with individual pathway and gene names found in Supplemental Table S1.

## Supplementary Material

dvae011_Supp

## Data Availability

Raw sequencing data were uploaded into the National Center for Biotechnology Information Gene Expression Omnibus database under accession number GSE255231.
