# Supplementary material for: Impaired energy expenditure following exposure to either DDT or DDE in mice may be mediated by DNA methylation changes in brown adipose
Source: Environ Epigenet. 2024 Aug 23;10(1):dvae011. doi: 10.1093/eep/dvae011 (PMC11472829; doi:10.1093/eep/dvae011)
Supplement: dvae011_Supp [file dvae011_supp.zip › suppl_data/Supplementary_Figures.doc]

**Supplementary Figures**

**Title**: Impaired energy expenditure following exposure to either DDT or DDE in mice may be mediated by DNA methylation changes in brown adipose

**Authors**: J.A. Jugan^1^, K.B. Jackson^1^, S.E. Elmore^1^, M.A. La Merrill^1^

**Affiliations**: ^1^University of California, Davis, Department of Environmental Toxicology

**Figure S1**. KEGG pathway for taurine and hypotaurine metabolism was significantly enriched in BAT of infant female mice perinatally exposed to DDT (left) and adult female mice perinatally exposed to DDE (right) where an increase in DNA methylation is represented by yellow and decrease is represented by blue.

**Figure S2**. KEGG pathway for adrenergic signaling in cardiomyocytes was significantly enriched in BAT of adult female mice perinatally exposed to DDT (left) or DDE (right) where an increase in RNA expression is represented by yellow and decrease is represented by blue.

**Figure S3**. KEGG pathway for the gap junction was significantly enriched in BAT of adult mice perinatally exposed to DDT where an increase in DNA methylation (left) or RNA expression (right) is represented by yellow and decrease is represented by blue.

**A**

**B**

**Figure S4**. KEGG pathway for (A) the lysosome and (B) retinol metabolism in animals was significantly enriched (p<0.05) in BAT of adult female mice perinatally exposed to DDT (left) or DDE (right) where an increase in DNA methylation is represented by yellow and decrease is represented by blue.

**A**

**B**

**Figure S5**. KEGG pathway for (A) nicotinate and nicotinamide metabolism and (B) glycosphingolipid biosynthesis – globo and isoglobo series was significantly enriched in BAT of adult female mice perinatally exposed to DDT (left) or DDE (right) where an increase in DNA methylation (left) or RNA expression (right) is represented by yellow and decrease is represented by blue.

**Figure S6**. Percent cell death in PAZ6 cells differentiated for 14 days in the presence of dimethyl sulfoxide vehicle (VEH), DDT, or DDE measured by trypan blue exclusion assay and repeated across three individual plates. Statistical analysis was performed using one-way ANOVA. There is no significant difference between VEH and DDX treatment groups. Datapoints shown represent individual values ±SEM.

**Figure S7**. PAZ6 cells at passage 13 (A) and passage 14 (B), imaged at day 0, day 4, and day 14 of differentiation. Neutral lipids are stained red with Oil Red O. Representative images were taken at 200X, scale bars represent 200 µm. Staining was repeated across two individual plates.

**Figure S8.** DNA expression of endogenous control, B2M, and Y chromosome-specific gene, SRY, in PAZ6 human brown adipocytes (grey), MDA-MB-231 human female breast adenocarcinoma cells (pink), and HEPG2 human male hepatocellular carcinoma cells (blue). Datapoints shown represent three technical replicates.
